# Development and Validation of a High-Performance Liquid Chromatography–Tandem Mass Spectrometry Method to Determine Promethazine and Its Metabolites in Edible Tissues of Swine

**DOI:** 10.3390/foods12112180

**Published:** 2023-05-29

**Authors:** Dehui Wen, Rong Shi, Haiming He, Rundong Chen, Yingzi Zhang, Rong Liu, Hong Chen

**Affiliations:** 1 National Reference Laboratory of Veterinary Drug Residues, Guangdong Provincial Key Laboratory of Veterinary Pharmaceutics Development and Safety Evaluation, College of Veterinary Medicine, South China Agricultural University, Guangzhou 510642, China; 20203073135@stu.scau.edu.cn (D.W.);; 2 Quality Supervision, Inspection and Testing Center for Domestic Animal Products (Guangzhou), Ministry of Agriculture and Rural Affairs, College of Veterinary Medicine, South China Agricultural University, Guangzhou 510642, China

**Keywords:** promethazine, promethazine sulfoxide, monodesmethyl-promethazine, swine edible tissues, high-performance liquid chromatography–tandem mass spectrometry

## Abstract

This study aimed to determine promethazine (PMZ) and its metabolites, promethazine sulfoxide (PMZSO) and monodesmethyl-promethazine (Nor_1_PMZ), in swine muscle, liver, kidney, and fat. A sample preparation method and high-performance liquid chromatography–tandem mass spectrometry (LC–MS/MS) analysis were established and validated. The samples were extracted using 0.1% formic acid–acetonitrile and purified with acetonitrile-saturated n-hexane. After concentration by rotary evaporation, the extract was re-dissolved in a mixture of 0.1% formic acid-water and acetonitrile (80:20, *v*/*v*). Analysis was performed using a Waters Symmetry C_18_ column (100 mm × 2.1 mm i.d., 3.5 μm) with 0.1% formic acid–water and acetonitrile as the mobile phase. The target compounds were determined using positive ion scan and multiple reaction monitoring. PMZ and Nor_1_PMZ were quantified with deuterated promethazine (PMZ-d6) as the internal standard, while PMZSO was quantified using the external standard method. In spiked muscle, liver, and kidney samples, the limits of detection (LOD) and limits of quantification (LOQ) for PMZ and PMZSO were 0.05 μg/kg and 0.1 μg/kg, respectively, while for Nor_1_PMZ, these values were 0.1 μg/kg and 0.5 μg/kg, respectively. For spiked fat samples, the LOD and LOQ for all three analytes were found to be 0.05 μg/kg and 0.1 μg/kg, respectively. The sensitivity of this proposed method reaches or exceeds that presented in previous reports. The analytes PMZ and PMZSO exhibited good linearity within the range of 0.1 μg/kg to 50 μg/kg, while Nor_1_PMZ showed good linearity within the range of 0.5 μg/kg to 50 μg/kg, with correlation coefficients (r) greater than 0.99. The average recoveries of the target analytes in the samples varied from 77% to 111%, with the precision fluctuating between 1.8% and 11%. This study developed, for the first time, an HPLC–MS/MS method for the determination of PMZ, PMZSO, and Nor_1_PMZ in four swine edible tissues, comprehensively covering the target tissues of monitoring object. The method is applicable for monitoring veterinary drug residues in animal-derived foods, ensuring food safety.

## 1. Introduction

Promethazine (PMZ) is a first-generation antihistamine drug known for its anti-allergic properties. It exhibits additional central inhibitory effects on the subcortical regions of the brain, resulting in significant central sedation, hypnotic, antiemetic, and antipyretic effects, making it commonly used for sedation and sleep [1,2,3]. In China, PMZ is approved for treating allergic reactions in animals such as sheep and pigs, including urticaria and serum sickness. Occasionally, a small number of farmers illegally use it in the breeding process of food animals in order to reduce animal movement, speed up weight gain, or reduce stress reactions during transportation [4]. 

There have been reports of adverse reactions due to PMZ abuse in humans, including drug-induced mental disorders and cardiovascular diseases in certain individuals [5,6,7]. However, the illegal use of PMZ in animal feed and breeding can also pose health hazards to consumers through drug residues in animal-derived foods and result in environmental pollution and other risks [8,9]. Chinese Ministry of Agriculture Announcements No. 176 and No. 2583 prohibit the use of promethazine hydrochloride in animal feed and drinking water. In March 2010, the Chinese Ministry of Health published the fourth batch of “non-food substances that may be illegally added to food and food additives that are easily abused” list, which included promethazine. Regulations in Japan, the United States, and the European Union also prohibit the residuals of thiazine tranquilizers and their metabolites in animal-derived foods. Furthermore, the use of PMZ formulations in food animals has not been approved in the European Union, the United States, and other countries and regions. The Ministry of Agriculture and Rural Affairs of China, in order to ensure the safety of animal-derived food and regulate the use of veterinary drugs, has arranged research projects which include PMZ residue studies. We were fortunate to participate in these research projects, to establish a detection method for PMZ and its metabolites in accordance with the Ministry of Agriculture and Rural Affairs of China’s No. 326 Announcement “Guiding Principles for Veterinary Drug Residue Elimination Tests” and the “Technical Guiding Principles for Quantitative Analysis Method Validation of Biological Samples”, released on 20 June 2022. In these technical guiding principles, experimental approaches, standards, parameters, and reference threshold values for detection method comply with the current international norms, such as COMMISSION IMPLEMENTING REGULATION (EU) 2021/808 of 22 March 2021.

According to previous reports, PMZ is primarily metabolized by CYP450 enzymes in animals [10,11,12]. Studies on PMZ metabolism in pig tissues seem to be scarce; no literature on PMZ metabolism in pigs was found. However, from the existing literature (see Table S1), PMZ metabolizes into five to eight metabolites, including PMZSO and Nor_1_PMZ in humans, rats, and mice. PMZSO and Nor_1_PMZ appear to be stable when present and account for a high proportion of metabolites which can be found in humans, rats, and mice. If drugs metabolize in mammals through CYP450 enzymes, there is a certain similarity in the metabolic pathways. Hence, we initially attempted to establish an LC–MS/MS analytical method for PMZ, PMZSO, and Nor_1_PMZ in pig plasma and tissues, then carried out a dosing trial in three experimental pigs. After a single intramuscular injection of PMZ, PMZ and its metabolites PMZSO and Nor_1_PMZ were found in the plasma of all three pigs. Ten days after the injection, PMZ, PMZSO, and Nor_1_PMZ were still present in plasma and tissue above the limit of quantification. Therefore, we eventually chose PMZ and its metabolites PMZSO and Nor_1_PMZ as the target analytes. Nor_1_PMZ was chosen as a target compound of analysis on drug residues in edible tissues for the first time in this study.

Various methods have been employed to detect PMZ, including enzyme-linked immunosorbent assay [13,14], spectroscopy [15,16,17], capillary electrophoresis [18,19], high performance liquid chromatography [20,21], gas chromatography–mass spectrometry [22,23,24,25,26,27,28,29], and liquid chromatography–tandem mass spectrometry (LC–MS/MS) [30,31,32,33,34,35,36,37,38,39,40,41,42,43]. However, most of the reported detection methods are used for PMZ formulations or detecting illegally added PMZ in animal feed [32,35,44,45]. Only a handful of methods have been developed to detect PMZ residues, or PMZ along with one of its metabolites, PMZSO, in animal-derived foods [14,21,33,37,41,43,44]. These methods are applicable to only certain edible tissues such as muscle, liver, and kidney. Notably, previous studies have not included fat tissue, which is an important animal source food. Therefore, in this study, fat tissue was included as a research object for the first time, considering its significance as an animal source food and as one of the target tissues for monitoring drug residues in food. The objective of this study was to establish a sample preparation and LC–MS/MS method for detecting PMZ and two of its metabolites in all edible tissues of swine, in order to provide technical support for monitoring PMZ and its metabolites in swine edible tissues, ensuring food safety.

## 2. Materials and Methods

### 2.1. Standards and Reagents

A 99.5% pure Promethazine Hydrochloride standard was procured from the China National Institute for Food and Drug Control, China. A Promethazine-d6 Hydrochloride standard with 98% chemical purity and 99.5% isotopic purity, a Promethazine Sulfoxide (PMZSO) standard with 96% purity, and a Monodesmethyl-Promethazine Hydrochloride standard with 97% purity were all sourced from Toronto Research Chemicals, Canada.

HPLC-grade acetonitrile (ACN) and methanol (MeOH) were obtained from Thermo Fisher Scientific, Waltham, MA, USA. HPLC-grade formic acid was bought from Shanghai Macklin Biochemical Co., Ltd., Shanghai, China. Analytical grade n-hexane was purchased from Tianjin Damao Chemical Reagent Factory, Tianjin, China. Ultrapure water was acquired from a Milli-Q water purification system (Millipore, Billerica, MA, USA).

### 2.2. Instruments and Equipment

Experiments utilized a high-performance liquid chromatography–tandem mass spectrometer (LC-30AD 220V liquid chromatograph, Shimadzu Corporation, Kyoto, Japan), equipped with an ESI5500 tandem quadrupole mass spectrometer and a Turbo Ionspray electrospray interface, as well as an Analyst 1.6.3 software workstation (Applied Biosystems, ABI, Corporation, MA, USA).

The chromatographic column employed was a Symmetry C_18_ (100 mm × 2.1 mm i.d., 3.5 µm) from Waters Corporation, Milford, MA, USA. A rotary evaporator (N-1300V-W, Tokyo Rikakikai Co., Ltd., Tokyo, Japan), a high-speed desktop centrifuge (LEGEND MACH 1.6R, Thermo Corporation, Waltham, MA, USA), and a vortex mixer (Vortex 3000, WIGGENS Co., Ltd., Straubenhardt, Germany) were also utilized. Nylon syringe filters, 13 mm, 0.22 µm, disposable, were sourced from Shanghai Ampu Company, Shanghai, China.

### 2.3. Preparation of Solution

Standard stock solution: Promethazine hydrochloride standard (calculated as PMZ, C_17_H_20_N_2_S), PMZSO standard (PMZSO, C_17_H_20_N_2_OS), and Monodesmethyl-Promethazine hydrochloride standard (calculated as Nor_1_PMZ, C_16_H_18_N_2_S) were accurately weighed and separately dissolved in HPLC-grade ACN in 50 mL volumetric flasks to achieve a concentration of 1000 μg/mL. These solutions were stored at −22 °C.

PMZ-d6 standard stock solution: A mass of 10 mg of promethazine-d6 hydrochloride standard (C_17_H_15_D_6_ClN_2_S) was transferred to a 10 mL volumetric flask, dissolved in HPLC-grade MeOH to achieve a concentration of 1000 μg/mL, sealed, and stored at −22 °C.

Mixed standard working solution: A volume of 1 mL of each of PMZ, PMZSO, and Nor_1_PMZ standard stock solutions were combined and diluted with HPLC-grade ACN to obtain series working solutions at concentrations of 2.5 μg/mL, 1.0 μg/mL, 0.5 μg/mL, 0.25 μg/mL, 0.05 μg/mL, 0.025 μg/mL, 0.005 μg/mL, and 0.0025 μg/mL. These solutions were stored at 4 °C.

PMZ-d6 working solution: An appropriate amount of PMZ-d6 standard stock solution was diluted with HPLC-grade ACN to obtain an internal standard solution to a final concentration of 1.0 μg/mL, sealed, and kept at 4 °C.

Acetonitrile saturated n-hexane: An appropriate amount of analytical grade n-hexane was added to an appropriate amount of ACN, mixed well, and allowed to stand until layered.

Formic Acid Solution (0.1%) in Water: A volume of 1.00 mL of HPLC-grade formic acid was transferred, diluted to 1 L volume with ultrapure water, and mixed well.

Formic Acid (0.1%) in Acetonitrile: A volume of 1.00 mL of HPLC-grade formic acid was transferred, and ACN was added to form a volume of 1 L. 

Formic Acid Solution (0.1%) in Water–Acetonitrile (80:20, *v*/*v*): A volume of 200 mL of HPLC-grade ACN was transferred to a 1 L volumetric cylinder, approximately 800 mL of 0.1% formic acid solution in water was added, and the combination was mixed well.

### 2.4. Chromatography and Mass Spectrometry Parameters

A Symmetry C_18_ (100 mm × 2.1 mm i.d., 3.5 µm) was used. The mobile phases were composed of phase A (0.1% formic acid solution in water) and phase B (acetonitrile, ACN). A flow rate of 0.3 mL/min was maintained with a gradient elution procedure, as presented in Table 1.

The mass spectrometer was operated in positive ion mode (ESI+), utilizing a multiple reaction monitoring (MRM) scan mode. The primary operating parameters are presented in Table 2.

The standard stock solution from Section 2.3 was diluted to 1 μg/mL with ACN and injected directly into the spectrometer for mass spectrometric optimization. Molecular ion peaks of the target analytes and the internal standard were identified by full-scan mass spectrometry in positive ion mode: m/z was 285.2 for PMZ, 301.3 for PMZSO, 271.3 for Nor_1_PMZ, and 291.3 for PMZ-d6. Each precursor ion underwent MS/MS scanning to determine and evaluate monitored ions for each analyte as a quantitative ion and a qualitative ion. The operation parameters for each ion were optimized using the mass spectrometric scan mode of multiple reaction monitoring (MRM). Consequently, the m/z of 86.2 and 198.1 were established as the quantitative and qualitative ions for PMZ, 198.2 and 239.1 for PMZSO, 197.9 and 240.3 for Nor_1_PMZ, and 92 and 240.3 for PMZ-d6. The quantitative and qualitative ion pairs, declustering potential, and collision energy for each target compound are listed in Table 3. For quantification, PMZ and Nor_1_PMZ utilized PMZ-d6 as the internal standard, while PMZSO employed an external standard method.

### 2.5. Sample Preparation

The blank matrix used in this study came from the muscles, liver, kidneys, and fat of several different pigs and was not mixed during the processing.

Approximately 500 g of muscle, liver, and kidney samples had connective tissue, blood vessels, and fat removed before being chopped into a uniform slurry using a homogenizer. About 5.0 g ± 0.1 g of this slurry was weighed into a 50 mL centrifuge tube, mixed with 100 μL of PMZ-d6 internal standard working solution (1 μg/mL), vortexed for 30 s, and left to stand for 30 min. After adding 10 mL of 0.1% formic acid in acetonitrile, the mixture was vortexed for 1 min and shaken for 10 min at 100% speed on a platform shaker before being centrifuged at 10,000 rpm for 10 min. The supernatant was transferred to a pear-shaped bottle. Another 10 mL of 0.1% formic acid acetonitrile was added to the residue in the centrifuge tube, and the above steps were repeated for a second extraction. Both extraction liquids were collected in a pear-shaped bottle for purification and concentration.

Around 500 g of subcutaneous fat from pig, free from muscle and connective tissue, was homogenized to produce a uniform slurry. About 5.0 ± 0.1 g of this fat slurry was weighed into a 50 mL centrifuge tube, into which 100 μL of PMZ-d6 internal standard working solution (1 μg/mL) were added, before being vortexed for 30 s and left to stand for 30 min. Then, 10 mL of acetonitrile saturated n-hexane was added, vortexed until the fat was completely dissolved, and left to stand for 30 min. After adding 10 mL of 0.1% formic acid in acetonitrile, the fat mixture was vortexed for 1 min and shaken for 10 min at 100% speed on a platform shaker, before being centrifuged at 10,000 rpm for 10 min. The upper hexane layer was discarded, and the lower extraction liquid was transferred to a new 50 mL centrifuge tube for purification. 

The extraction liquids of muscle, liver, kidney, and fat were added to 10 mL of acetonitrile-saturated n-hexane and vortexed for about 30 s to mix. After settling, the upper hexane layer was discarded and the lower extraction liquid was added to 10 mL of anhydrous ethanol. This was then reduced in volume by using a rotary evaporator at 45 °C, then 5 mL of 0.1% formic acid water–acetonitrile was added and vortexed for 30 s to dissolve the residue completely. After this, 5 mL of n-hexane-saturated acetonitrile was added to the solution and vortexed to mix, then left to stand for layering. Approximately 1 mL of the lower solution was transferred to a 1.5 mL centrifuge tube and centrifuged at 14,000 r/min, 0 °C, for 10 min. The clarified middle liquid was filtered using 0.22 μm nylon syringe filters, sealed in an autosampler vial, and stored at 4 °C for analysis.

### 2.6. Limit of Detection and Limit of Quantification

To establish the limit of detection (LOD) and limit of quantification (LOQ), a blank tissue sample homogenate (5 ± 0.1 g) was spiked with 100 μL of PMZ-d6 internal standard working solution (1 μg/mL) and 100 μL of mixed standard working solution. Thus, spiked samples at varying concentrations of 0.05 μg/kg, 0.1 μg/kg, 0.5 μg/kg, and 1 μg/kg were prepared. These samples were processed and analyzed by the method described in Section 2.4 and Section 2.5. The concentration of the sample with a signal-to-noise ratio (S/N) ≥ 3 was considered the LOD, and the concentration with an S/N ≥ 10 was regarded as the LOQ.

### 2.7. Calibration Curve and Linearity

Blank tissue sample slurries (5 ± 0.1 g) were spiked with a 1 μg/mL PMZ-d6 internal standard working solution (100 μL) and a mixed standard working solution (100 μL) to achieve varying concentrations—for PMZ and PMZSO, ranging from 0.1 μg/kg to 50 μg/kg; for Nor_1_PMZ, ranging from 0.5 μg/kg to 50 μg/kg. These samples were processed and analyzed by the method described in Section 2.4 and Section 2.5. The calibration curve and correlation coefficient (r) were determined using a weighted least-squares method with the ratio of the concentration of PMZ, Nor_1_PMZ, and PMZ-d6 as the abscissa and the peak area ratio of the quantitative ion pairs of PMZ, Nor_1_PMZ, and PMZ-d6 as the ordinate, with the weight chosen as 1/X^2^. The calibration curve and correlation coefficient of PMZSO were obtained using a weighted least-squares method with the concentration of PMZSO as the abscissa and the peak area of the PMZSO quantitative ion pair as the ordinate, with the weight chosen as 1/X^2^. The experiment was repeated in triplicate.

### 2.8. Recovery and Precision

To assess recovery and precision, blank tissue samples slurry (5 ± 0.1 g) were spiked with mixed standard working solutions of low, medium, and high concentration. These spiked samples at concentrations of 0.5 μg/kg, 5 μg/kg, and 50 μg/kg were processed and analyzed. The recovery and relative standard deviation (RSD) of the sample determination values were calculated, with RSD serving as an indicator of precision. The experiment was repeated for three batches to test inter-day precision.

### 2.9. Investigation of Matrix Effects

A homogenized blank tissue sample of 5 g ± 0.1 g, processed as delineated in Section 2.5, was utilized to generate a sample matrix solution. The mixed standard working solution from Section 2.3, amounting to 100 μL, was separately integrated into the sample matrix solution, thus forming matrix-matched samples at concentrations of 0.1 μg/kg, 0.5 μg/kg, 1 μg/kg, 5 μg/kg, 10 μg/kg, 20 μg/kg, and 50 μg/kg. These samples were analyzed by the method described in Section 2.4, and the matrix-matched sample curve was subsequently plotted. This experiment was conducted thrice.

The mixed standard working solution, described in Section 2.3, was diluted with methanol, resulting in concentrations of 0.1 μg/L, 0.5 μg/L, 1 μg/L, 5 μg/L, 10 μg/L, 20 μg/L, and 50 μg/L. The analysis was conducted as per the conditions specified in Section 2.4, enabling the derivation of the standard working solution curve.

The matrix effect, which refers to the extent of the sample matrix’s influence on target compound determination, was evaluated by comparing the slope of the matrix-matched sample curve with the standard working solution of equivalent concentration. Matrix enhancement is indicated by ME > 0, while ME < 0 signifies matrix suppression. Low signal interference from the matrix, which can be overlooked, occurs when 0 ≤ |ME| ≤ 20%. Moderate matrix interference is signaled by 20% < |ME| < 50%, and strong matrix interference is inferred when |ME| ≥ 50%. 

Matrix effect is calculated using the following formula:

(1) $$ME=\left(\frac{S_{m}}{S_{s}}-1\right)\times 100\%$$

ME: Matrix Effect;
*S_m_*: Slope of the curve of matrix-matched samples;
*S_s_*: Slope of the curve of standard working solution.

### 2.10. Stability Test

A homogenized blank tissue sample (5 ± 0.1 g), combined with a low or high concentration of the mixed standard working solution, was used to yield a quality control (QC) sample. QC samples, boasting target drug concentrations of 0.5 µg/kg and 50 µg/kg, were processed in accordance with the method delineated in Section 2.5. The stability of these samples was assessed at different situations: after 30 days of storage at −22 °C, after a week’s storage at 4 °C, after three freeze–thaw cycles, and after exposure to room temperature and light for 24 h. Each concentration was replicated thrice. The actual measured concentration was compared with the theoretical added concentration. The deviation between each concentration’s mean value and the theoretical concentration was calculated, with the relative standard deviation (RSD) aimed to be within 15%.

## 3. Results

### 3.1. Optimization of HPLC–MS/MS Conditions

**Figure 1:**
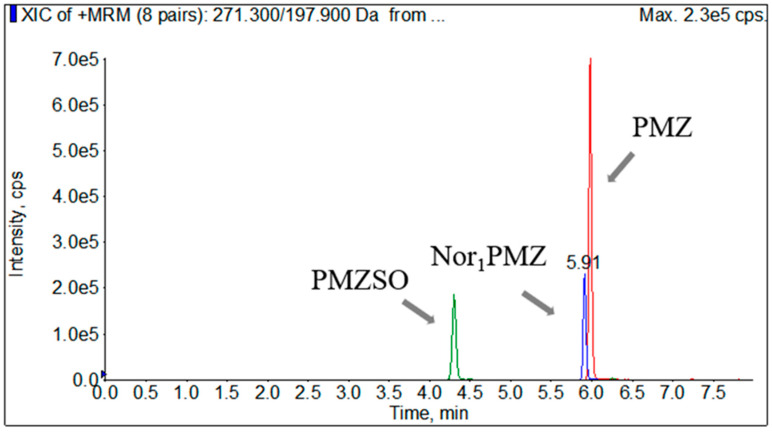
The characteristic ion mass spectrometry of the mixed standard working solution of 0.005 μg/mL.

The Symmetry C_18_ column (100 mm × 2.1 mm i.d., 3.5 µm), supplied by Waters, USA, was chosen for separation in this study. Several mobile phase combinations were tested, including 0.1% formic acid water–acetonitrile, 0.1% acetic acid water–acetonitrile, 0.2% formic acid water–acetonitrile, and a blend of 0.1% formic acid and 0.1% acetonitrile. The results indicated that the 0.1% formic acid water–acetonitrile mobile phase system provided the optimum response value and retention time. Figure 1 depicts the characteristic ion mass spectrometry of a mixed standard working solution of 0.005 μg/mL, with mobile phase of 0.1% formic acid in water and acetonitrile. 

**Figure 2:**
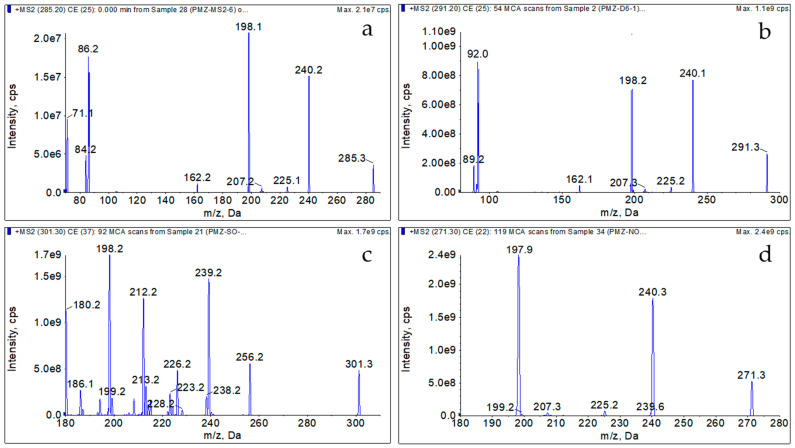
Representative MRM chromatograms of the precursor ions and primary product ions of the analytes and internal standard in the standard working solution: (**a**) PMZ, (**b**) PMZ-d6, (**c**) PMZSO, and (**d**) Nor_1_PMZ.

After the optimization of operational parameters, the molecular ions and the product ions of PMZ, PMZSO, Nor_1_PMZ, and PMZ-d6 in standard working solutions were scanned under suitable conditions, as illustrated in Figure 2.

### 3.2. Selection of Extraction Reagents

**Figure 3:**
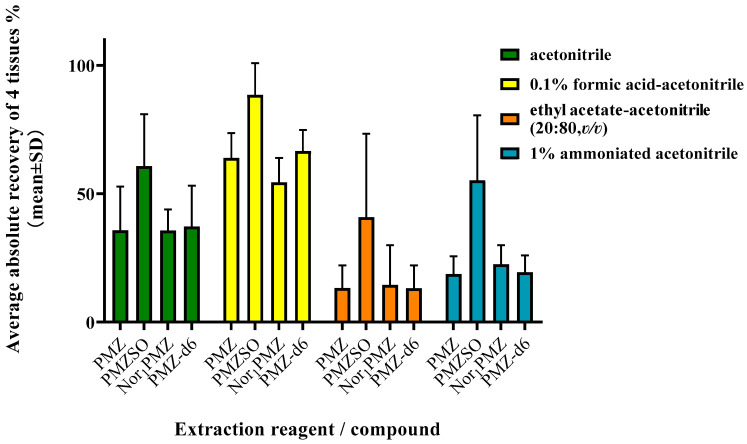
Average absolute recoveries of four analytes in four types of tissues in different extraction reagents.

The actual absolute recoveries of four analytes in muscle, liver, kidney, and fat tissue were compared using four extraction reagents: acetonitrile, 0.1% formic acid in acetonitrile, a blend of ethyl acetate and acetonitrile (20/80, *v*/*v*), and 1% ammoniated acetonitrile, as depicted in Figure 3. In Figure 3, the bar represents the average absolute recovery rate of each analyte in four types of tissues, extracted using different extraction reagents, and the error bar represents the standard deviation. The extraction efficiency of 0.1% formic acid in acetonitrile was superior to the others. Consequently, it was chosen as the extraction reagent for the four analytes. 

### 3.3. Methodological Validation

Selectivity was evaluated by comparing the chromatograms derived from spiked tissue samples and blank tissue samples, processed and detected following the method outlined in Section 2.4 and Section 2.5. It was demonstrated that no endogenous peaks from blank samples were present, and no interfering signals were observed at the retention times of each monitored ion of the analytes. As such, the method developed in this study allowed for accurate qualitative and quantitative analysis of PMZ and its metabolites, PMZSO and Nor_1_PMZ.

The limit of detection (LOD), limit of quantification (LOQ), linear range, and linearity were assessed using spiked samples. After processing and detecting the samples in accordance with Section 2.6, the LOD and LOQ for PMZ and PMZSO were determined to be 0.05 μg/kg and 0.1 μg/kg, respectively, in muscle, liver, and kidney samples; for Nor_1_PMZ, the LOD and LOQ were 0.1 μg/kg and 0.5 μg/kg, respectively. For spiked fat samples, the LOD and LOQ for all three analytes were found to be 0.05 μg/kg and 0.1 μg/kg, respectively. Employing the method described in Section 2.7, PMZ and PMZSO displayed good linear relationships in the range of 0.1 μg/kg to 50 μg/kg across the four tissue types. Nor_1_PMZ also exhibited a strong linear relationship in the range of 0.5 μg/kg to 50 μg/kg, with correlation coefficients (r) exceeding 0.99. Refer to Table 4 for additional details.

Recovery and precision were evaluated using spiked samples, following the methodology presented in Section 2.8. As can be seen in Table 5 (original data are shown in Table S2), average recoveries for PMZ, PMZSO, and Nor_1_PMZ in muscle, liver, kidney and fat ranged from 77% to 111%. The intra-day and inter-day precision for all tissues remained less than 15%, thereby meeting the Ministry of Agriculture and Rural Affairs of China’s technical guiding principles for residue analysis methods.

After processing and analyzing the samples as described in Section 2.9, the matrix effects were determined, as presented in Table 6. The matrix effects for the four types of tissue were predominantly negative, signifying a suppression effect on the signal of the compounds. The matrix effect on the three target compounds in pig fat tissue suggested weak matrix interference. In contrast, the matrix effect on the three target compounds in pig muscle and kidney tissues indicated moderate matrix interference. In pig liver tissue, the matrix effect on the three target compounds signified strong matrix interference. These findings underscore the necessity for thoughtful consideration of tissue matrix types when analyzing analytes, as varying matrices can influence the accuracy of the results. 

The stability of the samples was assessed following the methodology outlined in Section 2.10. As detailed in Table 7, the relative standard deviations (RSDs) for each analyte concentration within tissue samples, subjected to conditions such as ambient temperature and light exposure for 24 h, refrigeration at 4 °C for 48 h, a three-cycle freeze–thaw process, and prolonged storage for a month, typically hovered around ±15%. Hence, the structural and compositional stability of PMZ, PMZSO, and Nor_1_PMZ in tissue samples proved to be fairly robust under a range of conditions.

## 4. Discussion

Thiophene compounds encompass amino groups, which, when dissociated in water, exhibit alkalinity. These compounds may be adsorbed by residual silicon hydroxyl groups present on the surface of the stationary phase of a chromatographic column. To address this issue, the selection of fully end-capped C_18_, phenyl, and C_8_ chromatographic columns is recommended. The Symmetry C_18_ column (100 mm × 2.1 mm i.d., 3.5 µm, Waters, Milford, MA, USA) was utilized for separation in this investigation. In LC–MS/MS analysis, the ESI+ mode is favorable for alkaline PMZ and its metabolites, while acidic mobile phase systems tend to form [M + H] ^+^ ions. Acetonitrile and water, which are frequently used as mobile phases, can be proportioned according to specific requirements. Formic acid or acetic acid serve as typical protonation reagents in the LC–MS mobile phase. This study examined the effects of introducing different ratios of formic acid or acetic acid into the mobile phase. It was discovered that acetic acid increased the baseline of the Nor_1_PMZ representative ion chromatogram, rendering it unsuitable. The most optimal retention time and representative ion chromatograms for the analytes were achieved by adding 0.1% (by volume) formic acid to the aqueous phase.

Matrix effects from animal tissue samples can interfere with the accuracy of drug content analysis in tissues. The internal standard method is routinely employed to mitigate matrix effects and significantly enhance the accuracy and precision of the analysis. Numerous studies have reported the use of the internal standard method in determining PMZ and its metabolite content. Metronidazole served as the internal standard for estimating PMZ and PMZSO in rat plasma and various tissues [36]. PMZ-d6 and PMZSO-d6 were utilized as internal standards to detect the content of PMZ and PMZSO in pig muscle, liver, and kidney [43]. Donepezil was used to detect drugs, include PMZ, in human plasma and urine [31]. Haloperidol was reported to be used as internal standard to quantify chlorpromazine and PMZ in pig kidneys [21], and loratadine was used as internal standard when studying PMZ and ephedrine mixture [38]. The PMZ-d6, a deuterated isotope of PMZ, was employed as the internal standard for quantification in this study. 

In the research work, it was found that PMZ, PMZSO, Nor_1_-PMZ, and PMZ-d6 stock solutions and working solutions were stable long-term at −20 °C and 4 °C, and were stable at room temperature and during the sample preparation process. However, after evaporating the solvent of PMZ-d6 working solution, the response value of PMZ-d6 detected by LC–MS/MS significantly decreased after one week at room temperature and exposed to the air. Therefore, we sealed and stored the solution containing PMZ-d6 in the refrigerator. After the solvent of the sample solution containing PMZ-d6 is evaporated by a rotary evaporator, it should be immediately re-dissolved, sealed, and stored at 4 °C.

It was found that the recovery of PMZSO was generally significantly high (>120%) while quantified by PMZ-d6 with internal standard method, though the recovery of PMZ and Nor_1_PMZ was in the range of 80–120%. After investigations, it was found that, in spiked samples, the actual extraction recovery of PMZ, PMZ-d6, and Nor_1_PMZ were all between 60% and 70%, which were very close. However, the actual extraction recovery of PMZSO was above 85%, which was significantly different from the internal standard and other analytes, as shown in Figure 3. As such, PMZ-d6 is unsuitable for quantification analysis of PMZSO. As a metabolite, PMZSO shows stronger polarity than PMZ, with its chemical properties differing from those of PMZ, PMZ-d6, and Nor_1_PMZ. Finally, the internal standard method was used for quantifying PMZ and Nor_1_PMZ, and the external standard method was used for quantifying PMZSO.

Based on the physicochemical characteristics of the target analytes in this study, along with evidence from previous studies [33,41,46], several extraction solvents, including ACN, 0.1% formic acid in ACN, ethyl acetate—ACN (20:80, *v*/*v*), and 1% ammoniated ACN, were investigated for their extraction recovery efficacy in pig tissues. Results indicated that formic acid–acetonitrile combination exhibited the most efficient extraction recovery across all analytes, as depicted in Figure 3. Considering the efficiency of extraction for PMZ, PMZSO, Nor_1_PMZ, and PMZ-d6 across four tissue samples, 0.1% formic acid in ACN was utilized as the extraction solvent for this study. It was observed that the extraction efficiency could be boosted by adding a slight amount of acid. However, with an increasing increment in formic acid volume, the extraction liquid for liver and kidney became darker, harboring more impurities, and, thus, posing interference in instrument detection. Consequently, an optimal extractant ratio of 0.1% formic acid in ACN was established.

Fat tissue is a significant animal source food and one of the target tissues for monitoring drug residues. However, fat samples pose challenges in sample preparation and detection procedures due to their high lipophilic impurity content. The extraction recovery of analytes in fat is generally low. In this study, various procedures were explored to enhance extraction and purification efficacy in fat samples. It was determined that complete dissolution of the fat sample slurry in n-hexane prior to analyte extraction improved extraction recovery. During the sample concentration and purification process, the lipid-rich impurity content in the sample solvent could be discarded through extraction with n-hexane, both before and after the extraction solvent was removed using a rotary evaporator. Centrifuging the sample solution at 0 °C or lower facilitated the upward migration of lipid-interfering substances. Finally, the parameters of the fat tissue detection method complied with the requirements of technical guiding principles.

## 5. Conclusions

We have, for the first time, developed and validated an LC–MS/MS method for the determination of promethazine and its two metabolites across all edible tissues of swine, in accordance with the Ministry of Agriculture and Rural Affairs of China’s technical guiding principles. In this method, 0.1% formic acid in acetonitrile served as the extraction solvent, and LC–MS/MS was used for analyte detection. The limit of quantification ranged between 0.1 μg/kg and 0.5 μg/kg, demonstrating a sensitivity equal to or surpassing previous reports. This study included swine fat as a research subject for the first time and Nor_1_-PMZ as one of the target analytes, thereby presenting an accurate and reliable detection method for monitoring PMZ residues and its metabolites in swine edible tissues.

## Figures and Tables

**Table 1:** Gradient program.

Time (min)	Phase B (%)	Phase A (%)	Flow Rate (μL/min)
1.5	10	90	300
6.7	50	50	300
7.0	10	90	300
8	10	90	300

**Table 2:** Operating parameters for mass spectrometer.

Parameter	Condition
The source temperature, TEM	550 °C
Collision Gas, CAD	8 psi
Curtain gas, CUR	40 psi
Nebulizer gas1, GS1	55 psi
Ionspray, IS	5500 V
Sheath gas (N_2_) flow	25 arbitrary units
Entrance Potential, EP	10 V
Collision Cell Exit Potertial, CXP	18 V
Ion source gas2, GS2	55 psi
Dwell time, DT	50 ms

**Table 3:** Qualitative and quantitative ion pairs, declustering voltage, collision energy, and retention time for analytes and internal standard.

Analyte	Precursor Ion (*m*/*z*)	Product Ions (*m*/*z*)	Declustering Voltage (v)	Collision Energies (Ev)	Retention Time (min)
PMZ	285.2	86.2 */198.1	60	25/33	5.99
PMZSO	301.3	198.2 */239.1	60	51/31	4.30
Nor_1_PMZ	271.3	197.9 */240.3	60	35/20	5.91
PMZ-d6	291.3	92 */240.1	60	27/20	5.99

Note: The sub ions marked with “*” are the quantification ions.

**Table 4:** Linear equations, correlation coefficient (r), limit of detection (LOD), and limit of quantification (LOQ) of PMZ and its two metabolites.

Tissues	Analyte	Linear Range	Regression Equation *	r	LOD (μg/kg)	LOQ (μg/kg)
Muscle	PMZ	0.1–50 μg/kg	Y = 2.43x − 0.0116	0.9993	0.05	0.1
	PMZSO		Y = 1.49x + 0.00797	0.9977		
	Nor_1_PMZ	0.5–50 μg/kg	Y = 1.19x − 0.00557	0.9993	0.1	0.5
Liver	PMZ	0.1–50 μg/kg	Y = 2.82x − 0.062	0.9992	0.05	0.1
	PMZSO		Y = 2.79x + 0.0583	0.9992		
	Nor_1_PMZ	0.5–50 μg/kg	Y = 1.19x − 0.0316	0.9996	0.1	0.5
Kidney	PMZ	0.1–50 μg/kg	Y = 2.22x + 0.0125	0.9997	0.05	0.1
	PMZSO		Y = 1.53x + 0.022	0.9972		
	Nor_1_PMZ	0.5–50 μg/kg	Y = 0.812x + 0.00942	0.9988	0.1	0.5
Fat	PMZ	0.1–50 μg/kg	Y = 1.38x + 0.0595	0.9990	0.05	0.1
	PMZSO		Y = 0.583x + 0.0102	0.9996		
	Nor_1_PMZ		Y = 0.926x + 0000146	0.9985		

* Y: peak area of analyte, x: concentration of analyte.

**Table 5:** Recovery and precision of spiked blank samples.

Tissues	Analyte	Concentration (μg/kg)		Recovery (%, $\bar{X}$, n = 6)	Intra-Day RSD (%, n = 6)			Inter-Day RSD (%)
		Spiked	Found ($\bar{X}$, n = 6)		Batch I	Batch II	Batch III	
Muscle	PMZ	0.1	0.077~0.082	77~82	7.1	11	11	9.9
		5	4.72~5.00	92~100	2.8	3.2	4.8	5.1
		50	47.15~52.33	94~105	5.7	3.8	3.8	6.1
	PMZSO	0.1	0.099~0.10	99~103	7.0	8.1	10	8.3
		5	4.64~5.31	93~106	2.1	3.0	7.7	7.6
		50	43.12~45.23	86~90	2.4	3.3	3.2	3.5
	Nor_1_PMZ	0.5	0.45~0.52	90~104	7.6	8.4	9.4	10
		5	4.75~5.04	95~101	6.5	3.6	4.9	5.5
		50	45.92~55.58	92~111	3.2	3.3	6.0	9.3
Liver	PMZ	0.1	0.097~0.1	97~104	11	7.1	6.3	8.4
		5	4.35~5.37	87~107	2.3	8.0	5.2	10
		50	44.37~45.42	89~91	5.0	5.6	4.4	4.8
	PMZSO	0.1	0.098~0.1	98~101	8.1	5.1	9.8	7.6
		5	4.54~5.01	91~100	6.6	3.9	5.5	6.9
		50	48.23~52.20	96~104	4.4	5.3	1.8	5.1
	Nor_1_PMZ	0.5	0.47~0.49	94~97	8.6	6.9	6.4	7.5
		5	4.57~4.93	91~98	5.5	9.4	7.0	7.8
		50	47.32~50.40	95~101	4.1	4.4	3.6	4.6
Kidney	PMZ	0.1	0.086~0.094	86~94	5.0	2.9	7.8	6.7
		5	4.22~4.48	84~90	5.9	4.8	4.3	5.4
		50	44.78~45.88	90~92	3.0	6.3	1.8	4.0
	PMZSO	0.1	0.095~0.10	95~100	10	11	6.8	9.1
		5	5.02~5.23	101~105	4.3	5.6	3.1	4.6
		50	45.47~49.58	91~99	1.8	4.0	2.0	4.6
	Nor_1_PMZ	0.5	0.47~0.50	94~99	6.8	12	5.4	8.6
		5	4.93~5.22	97~104	7.1	11	5.9	7.9
		50	47.95~52.18	96~104	5.6	7.2	2.7	6.3
Fat	PMZ	0.1	0.096~0.10	96~100	7.6	7.6	9.5	7.6
		5	4.88~5.20	98~105	3. 7	3.1	3.2	4.1
		50	48.83~52.35	98~101	6.6	5.6	5.6	6.4
	PMZSO	0.1	0.094~0.10	95~101	7.2	10	9.0	9.1
		5	4.23~4.89	85~98	4.6	4.2	5.3	7.7
		50	45.48~46.40	91~93	3.6	5.1	3.2	4.8
	Nor_1_PMZ	0.1	0.096~0.10	96~101	9.9	9.0	11	9.7
		5	5.00~5.26	100~105	6.5	7.4	5.2	6.4
		50	43.43~49.38	87~99	5.0	7.7	7.9	7.5

**Table 6:** Matrix effects (%) of PMZ and its metabolites in four tissue types (n = 3).

Matrix	PMZ	SD (%)	PMZSO	SD (%)	Nor_1_PMZ	SD (%)
Muscle	−34.15	1.52	−24.94	2.05	−36.05	1.05
Liver	−56.68	0.86	−50.77	2.27	−49.15	1.01
Kidney	−19.75	1.91	−27.55	2.76	−22.96	2.71
Fat	−9.14	1.63	5.14	1.65	−0.14	1.86

**Matrix**

**PMZ**

**SD (** **%)**

**PMZSO**

**SD (** **%)**

**Nor_1_PMZ**

**SD (** **%)**

**Table 7:** Stability investigation of target compounds in various tissues, RSDs (%, n = 3).

Analyte	Tissues	Liver		Kidney		Fat		Muscle	
	Spiked (µg/kg)	0.5	50	0.5	50	0.5	50	0.5	50
Content detected of PMZ after treated	Room temperature and light for 24 h	9.06	9.54	5.71	9.54	4.21	5.44	8.68	4.68
	Stored at 4 °C for 48 h	5.96	6.68	8.70	6.81	7.27	7.32	8.23	4.92
	Stored at −22 °C for 30 days	11.80	13.47	15.06	13.47	5.15	6.72	11.98	8.11
	Repeated freeze–thawing 3 times	5.75	8.34	7.73	8.34	7.79	5.69	7.72	9.87
Content detected of PMZSO after treated	Room temperature and light for 24 h	9.66	9.69	6.43	5.86	5.92	7.63	10.42	6.05
	Stored at 4 °C for 48 h	10.68	8.23	6.12	3.59	7.84	6.67	7.28	3.96
	Stored at −22 °C for 30 days	11.71	9.64	11.89	10.56	8.85	8.95	12.86	13.62
	Repeated freeze–thawing 3 times	9.58	11.88	6.66	14.79	5.52	8.44	8.48	13.99
Content detected of Nor_1_PMZ after treated	Room temperature and light for 24 h	9.12	8.09	8.76	4.50	3.78	6.38	8.17	7.25
	Stored at 4 °C for 48 h	11.06	9.14	7.77	7.44	6.12	2.30	7.34	2.06
	Stored at −22 °C for 30 days	10.19	12.55	13.48	11.21	14.19	10.44	12.16	13.83
	Repeated freeze–thawing 3 times	13.48	6.70	7.45	10.04	6.64	7.24	7.20	11.85

## Data Availability

There were no publicly archived datasets created during the study.

## Supplementary Materials

The following supporting information can be downloaded at: https://www.mdpi.com/article/10.3390/foods12112180/s1, Table S1. Reports of analysis methods on PMZ and its metabolites in edible animal tissues [21,33,34,41,43,47,48,49,50,51,52]; Table S2. Original Data of Recovery and precision of spiked blank samples.

## References

[1] Liu Y. (2007). Solution Structure of Chlorpromazine Hydrochloride and Promethazine Hydrochloride and Electrochemical Performance of Their Interaction with DNA.

[2] Alyami H.S., Ibrahim M.A., Alyami M.H., Dahmash E.Z., Almeanazel O.T., Algahtani T.S., Alanazi F., Alshora D.H. (2021). Formulation of sublingual promethazine hydrochloride tablets for rapid relief of motion sickness. Saudi Pharm. J..

[3] Wang J., Lu C., Liu X., Zhang G., Zhang J., Gao M., Liu D., Zhang X., Liu Y. (2023). Histamine H1 receptor antagonist attenuates catecholamine surge and organ injury after severe burns. Front. Endocrinol..

[4] Gao Q. (2016). Determination of Phenothiazine Sedatives, Neonicotinic Insecticides, and Amide Herbicides in Food by LC-MS/MS.

[5] Abeysundera H., Craig B., Pullich Z. (2021). Promethazine-induced delirium with perceptual abnormalities: Are we thinking broadly when assessing patients?. BMJ Case Rep..

[6] Chiappini S., Schifano F., Corkery J.M., Guirguis A. (2021). Beyond the ‘purple drank’: Study of promethazine abuse according to the European Medicines Agency adverse drug reaction reports. J. Psychopharmacol..

[7] Adie S., Ingebrigtson M., Hamilton D., Tam M. (2021). A Case Of Promethazine-Induced Polymorphic Ventricular Tachycardia: Conduction Down The Tankfrom Consuming “Purple Drank”. J. Am. Coll. Cardiol..

[8] Ignoto S., Pecoraro R., Scalisi E.M., Buttigè S.E., Contino M., Ferruggia G., Salvaggio A., Brundo M.V. (2022). Acute Toxicity of a Marine Emerging Pollutant (Promethazine Hydrochloride) on *Artemia* sp. ACS Omega.

[9] Zhou Y., Hua X. (2021). The hazard and status quo of veterinary drug residues in animal derived food in China. Grain Oil.

[10] Bornschein I., Pfeifer S. (1979). Biotransformation of promethazine (Prothazin). Die Pharm..

[11] Bornschein I., Pfeifer S. (1980). Further sulfone metabolites of promethazine (Prothazin). Die Pharm..

[12] Ramanathan R., Geary R.S., Bourne D.W., Putcha L. (1998). Bioavailability of intranasal promethazine dosage forms in dogs. Pharmacol. Res..

[13] Gao B.L., Liu J., Dong L.X., Zhang L., Qin J.H., Wang J.P. (2014). Broad specific enzyme-linked immunosorbent assay for determination of residual phenothiazine drugs in swine tissues. Anal. Biochem..

[14] Shi F.S., Liu J., Zhang L., Liu J.X., Wang J.P. (2016). Development of an enzyme linked immunosorbent assay for the determination of phenothiazine drugs in meat and animal feeds. J. Environ. Sci. Heal. Part B.

[15] WU Y., Xing L., XU Y. (2008). Determination of promethazine and its metabolite in Urine by solid-phase extraction with celite and UV derivative spectrophotometry. Chin. J. Forensic Med..

[16] Zhang S.R., Yang J.D. (2009). Simultaneous Determination of Chlorpromazine Hydrochloride and Promethazine Hydrochloride by Resonance Rayleigh Scattering Spectra. J. Instrum. Anal..

[17] Yang J., Yang Q., Zou S., Wu L., Zhu Q. (2013). Simultaneous determination of chlorpromazine hydrochloride and promethazine hydrochloride using near-infrared spectroscopy. J. Anal. Sci..

[18] Wang L., Yang X., Mo J. (1999). Determination of Epinephrine, Chlorpromazine and Promethazine by Capillary Electrophoresis with Scanning Voltammetric Detector. Chin. J. Chromatogr..

[19] Li X., Yang Y., Zhou K. (2012). Simultaneous determination of chlorpromazine, promethazine, and their main metabolites by capillary electrophoresis electrophoresis with electrochemiluminescence. Chin. J. Chromatogr..

[20] Vanapalli S.R., Kambhampati S.P., Putcha L., Bourne D.W. (2001). A liquid chromatographic method for the simultaneous determination of promethazine and three of its metabolites in plasma using electrochemical and UV detectors. J. Chromatogr. Sci..

[21] Chen P., Fan S., Huang L., Yuan Z. (2005). Establishment of detection method for chlorpromazine and promethazine residues in swine kidney. Chin. J. Vet. Med..

[22] Hui-Qin W., Yong-Chun J., Ming-Zha C., Xiao-Lao H., Zhi-Xin Z. (2007). Simultaneous determination of 10 mental drugs by gas chromatography-mass spectrometry. Chin. J. Anal. Chem..

[23] Huang K., Zhu D., Li H., Ling C., Li L., Liu X. (2010). Analysis of promethazine and its metabolites in rat urine by GC-MS. J. Instrum. Anal..

[24] Cheng L., Zhang Y., Shen J., Wu C., Zhang S. (2009). GC–MS Method for Simultaneous Determination of Four Sedative Hypnotic Residues in Swine Tissues. Chromatographia.

[25] Tapadia K., Shrivas K., Upadhyay L.S.B. (2011). GC–MS Coupled with Hollow-Fiber Drop-to-Drop Solvent Microextraction for Determination of Antidepressants Drugs in Human Blood Sample. Chromatographia.

[26] Li W., Lin D., Sun H., Mutailifu M., Wang L. (2014). ASE-GC /MS analysis of common sedative hypnotic drugs in blood. Chin. J. Forensic Med..

[27] Li W., Li X., Lin D., Sun H., Shao K. (2018). Analysis of Five Hypnotic Sedative Drugs in Blood by Gas Chromatography—Mass Spectrometry with Supported Liquid Extraction. Anal. Test. Technol. Instrum..

[28] Zhao S.R.D. (2020). Determination of Four Sedative-hypnotic Drugs with GC/MS for a Case of Abnormal Death. Forensic Sci. Technol..

[29] Rosenberger W., Teske J., Klintschar M., Dziadosz M. (2022). Detection of pharmaceuticals in “dirty sprite” using gas chromatography and mass spectrometry. Drug Test. Anal..

[30] Liang Q., Qu J., Luo G., Wang Y. (2006). Rapid and reliable determination of illegal adulterant in herbal medicines and dietary supplements by LC/MS/MS. J. Pharm. Biomed. Anal..

[31] Liu P., Liang S., Wang B.-J., Guo R.-C. (2009). Development and validation of a sensitive LC-MS method for the determination of Promethazine hydrochloride in human plasma and urine. Eur. J. Drug Metab. Pharmacokinet..

[32] Suo D.-C., Zhao G.-L., Li L., Su X.-O. (2010). Simultaneous Determination of Seven Mental Drugs in Feeds by Liquid Chromatography-Tandem Mass Spectrometry. Chin. J. Anal. Chem..

[33] Li C., Yue Z., Zhao F., Hua H., Han R., Li L. (2010). Study on the Determination of Phenothiazide Residues in Aquatic Products by Liquid Chromatography Tandem Mass Spectrometry. Chin. J. Vet. Med..

[34] He L., Wang J., Zhang G., Liu R., Fang B. (2012). Simultaneous Determination of Tranquilizers and Carazolol Residues in Swine Tissues by Liquid Chromatography-Tandem Mass Spectrometry. Anal. Lett..

[35] Suo D.C., Zhao G.L., Wang P.L., Su X.O. (2014). Simultaneous Determination of beta-Agonists and Psychiatric Drugs in Feeds by LC-MS-MS. J. Chromatogr. Sci..

[36] Liang L. (2015). Application of Physiologically Based Pharmacokinetic Models for Assessing the Disposition of Promethazine in Simulated Weightless Rats.

[37] Cheng L., Shen J., Zhang Q., Zhang Y., Zhang S. (2014). Simultaneous Determination of Three Tranquillizers in Lamb Liver by Ultra-Performance Liquid Chromatography–Tandem Mass Spectrometry. Food Anal. Methods.

[38] Lin G. (2018). Pharmacokinetic study of promethazine ephedrine combination in rats under the simulated microgravity condition. J. Beijing Univ. Technol..

[39] Gao S., Zhou X., Lang L., Liu H., Li J., Li H., Wei S., Wang D., Xu Z., Cai H. (2019). Simultaneous Determination of Schisandrin and Promethazine with Its Metabolite in Rat Plasma by HPLC-MS/MS and Its Application to a Pharmacokinetic Study. Int. J. Anal. Chem..

[40] Proença P., Monteiro C., Mustra C., Claro A., Franco J., Corte-Real F. (2020). Identification and Quantification of Antipsychotics in Blood Samples by LC–MS-MS: Case Reports and Data from Three Years of Routine Analysis. J. Anal. Toxicol..

[41] Wang J., Ye J., Wang X., Zhong S., Chen Q. (2020). Determination of 15 sedative drug residues in livestock and poultry meat by ultra-high performance liquid chromatography tandem mass spectrometry. Farm Prod. Process..

[42] Fan L., An J., Cui Y., Dong Z. (2021). Development, validation, and application of a simple UPLC-MS/MS method for simultaneous quantification of five traditional antipsychotics in human plasma. Biomed. Chromatogr..

[43] Chen X., Zhou J., Jin M. (2022). Determination of 4 chlorpromazine and promethazine and their metabolites in swine tissues by liquid chromatography-tandem mass spectrometry with isotope internal standard dilution technique. J. Hyg. Res..

[44] Qi L., Duan L.-M., Sun X.-H., Zhang J., Zhang Z.-Q. (2015). Simultaneous determination of three banned psychiatric drugs in pig feed and tissue using solid-phase reactor on-line oxidizing and HPLC-fluorescence detection. Biomed. Chromatogr..

[45] Cunha R.R., Ribeiro M.M., Muñoz R.A., Richter E.M. (2021). Fast determination of codeine, orphenadrine, promethazine, scopolamine, tramadol, and paracetamol in pharmaceutical formulations by capillary electrophoresis. J. Sep. Sci..

[46] Huang M., Gao J.-Y., Zhai Z.-G., Liang Q.-L., Wang Y.-M., Bai Y.-Q., Luo G.-A. (2012). An HPLC–ESI-MS method for simultaneous determination of fourteen metabolites of promethazine and caffeine and its application to pharmacokinetic study of the combination therapy against motion sickness. J. Pharm. Biomed. Anal..

[47] Li R., Yang L., Zhang P., Luo Y., Zhang P., Gao Y. (2018). Rapid screening of 24 tranquillizer drugs in fish and fishery products by ultra-high performance liquid hromatographyquadrupole /electrostatic field orbitrap high resolution mass spectroscopy. Chin. J. Chromatogr..

[48] Wang K., Cao Q., Zhao L., Chen L., Gao Q., Yang L. (2020). Determination of Promethazine and Chlorpromazine in Poultry Eggs by High Performance Liquid Chromatography Tandem Mass Spectrometry. Food Ind..

[49] Sun L., Zhang L., Xu Q., Wang S., Wang X. (2010). Determination of ten sedative residues in pork and kidney by ultra performance liquid chromatography- tandem mass spectrometry. Chin. J. Chromatogr..

[50] Li Z., Li H., Ma Y., Wang S., Ren N., Guo W., Guo C., Li Y. (2019). High Performance Liquid Chromatography-Tandem Mass Spectrometry Detection of Promethazine in Animal-Derived Foods. Food Sci..

[51] Wang J., Ye J., Zhong S., Wang X., Chen Q. (2021). Determination of Antihistamine Residues in Livestock and Poultry Products by Ultra Performance Liquid Chromatography–Quadrupole–Time of Flight Mass Spectrometry. Sci. Technol. Food Ind..

[52] Liang Y., Li Y., Guo X., Li R., Gao L., Wang J., Fu G., Wan Y. (2016). Determination of chlorpromazine, promethazine, and vancomycin in pork product by liquid chromatography-mass spectrometry. Cereal Feed Ind..

