# Supplementary material for: Development and Validation of a High-Performance Liquid Chromatography–Tandem Mass Spectrometry Method to Determine Promethazine and Its Metabolites in Edible Tissues of Swine
Source: Foods. 2023 May 29;12(11):2180. doi: 10.3390/foods12112180 (PMC10252443; doi:10.3390/foods12112180)
Supplement: Supplementary file 1 [file foods-12-02180-s001.zip › foods-2387880 - Supplementary MaterialsV2.pdf]

**Table S1.** Reports of analysis methods on PMZ and its metabolites in edible animal tissues.

| **Analytes** | **Target tissue** | **Equipment** | **LOD, LOQ** | Reference |
|---|---|---|---|---|
| PMZ, PMZSO, Nor1PMZ | Swine muscle, liver, kidney, fat | LC-MS/MS | LOD: muscle, liver, and kidney 0.05 μg/kg for PMZ, PMZSO, 0.1μg/kg for Nor1PMZ. LOQ: muscle, liver, and kidney 0.1 μg/kg for PMZ and PMZSO, 0.5 μg/kg for Nor1PMZ. LOD and LOQ of fat, 0.05 μg/kg and 0.1 μg/kg for all analytes. | This study |
| 5 phenothiazines, including PMZ | Fish | LC-MS/MS | LOD：0.1ng/g LOQ：0.5ng/g | Li et al., 2010 [33] |
| 24 tranquillizer drugs, including PMZ | fish and fishery product | UPLC-Q-Orbitrap HRMS | LOQ：0.5μg/kg | Gao et al., 2018 [47] |
| PMZ, Chlorpromazine | Poultry Eggs | LC-MS/MS | LOD： for PMZ 0.05μg/kg，LOQ：for PMZ 0.17μg/k | Yang et al., 2020 [48] |
| 10 sedatives, including PMZ | Pork, kidney | UPLC-MS/MS | LOD：0.5μg/kg LOQ：1μg/kg | Sun et al.,2010 [49] |
| Tranquilizers, including PMZ | Swine muscle, liver, and kidney | LC-MS/MS | LOD：0.06-0.1g/kg LOQ：0.2-0.41g/kg | He et al., 2012 [34] |
| PMZ | Meat of bovine, porcine and ovine | LC-MS/MS | LOD：0.1μg/kg LOQ：0.3μg/kg | Li et al., 2019 [50] |
| 15 sedatives, including PMZ | Meat of bovine and poultry, porcine liver | UPLC-MS/MS | LOD：1μg/kg；LOQ：3μg/kg | Wang et al., 2020 [41] |
| PMZ, PMZSO | Meat of bovine and poultry, porcine liver | UPLC-Q-TOF/MS | LOD：PMZ 2μg/kg, PMZSO 0.5μg/kg LOQ： PMZ 6μg/kg, PMZSO 1.5 μg/kg | Wang et al., 2021 [51] |
| PMZ | Pork product | LC-MS/MS | LOD：PMZ 0.3ng/g, LOQ：PMZ 1 ng/g | Liang et al., 2016 [52] |
| PMZ, PMZSO | Porcine meat, liver, kidney | LC-MS/MS | LOD：PMZ 0.04μg/kg, PMZSO 0.17μg/kg LOQ：PMZ 0.12μg/kg, PMZSO 0.51μg/kg | Chen et al., 2022 [43] |

| | | | | |
|---|---|---|---|---|
| PMZ, Chlorpromazine | Porcine kidney | HPLC-UV | LOD: PMZ and Chlorpromazine 10μg/kg | Fan et al., 2005 [21] |

**References**


21. Chen, P.; Fan, S.; Huang, L.; Yuan, Z. Establishment of detection method for chlorpromazine and promethazine residues in swine kidney. *Chin. J. Vet. Med.* **2005**, *4*, 412–413, 416.

33. Li, C.; Yue, Z.; Zhao, F.; Hua, H.; Han, R.; Li, L. Study on the Determination of Phenothiazide Residues in Aquatic Products by Liquid Chromatography Tandem Mass Spectrometry. *Chin. J. Vet. Med.* **2010**, *3*, 384–387, 392.

34. He, L.; Wang, J.; Zhang, G.; Liu, R.; Fang, B. Simultaneous Determination of Tranquilizers and Carazolol Residues in Swine Tissues by Liquid Chromatography-Tandem Mass Spectrometry. *Anal. Lett.* **2012**, *45*, 1377–1389.

41. Wang, j.; Ye, J.; Wang, X.; Zhong, S.; Chen, Q. Determination of 15 sedative drug residues in livestock and poultry meat by ultra-high performance liquid chromatography tandem mass spectrometry. *Farm Prod. Process.* **2020**, *7*, 45–49.

43. Chen, X.; Zhou, J.; Jin, M. Determination of 4 chlorpromazine and promethazine and their metabolites in swine tissues by liquid chromatography-tandem mass spectrometry with isotope internal standard dilution technique. *J. Hyg. Res.* **2022**, *51*, 476–482.

47. Li, R.; Yang, L.; Zhang, P.; Luo, Y.; Zhang, P.; Gao, Y. Rapid screening of 24 tranquillizer drugs in fish and fishery products by ultra-high performance liquid hromatographyquadrupole /electrostatic field orbitrap high resolution mass spectroscopy. *Chin. J. of Chromatogr.*, **2018,** *36*, 125–135.

48. Wang, K.; Cao, Q.; Zhao, L.; Chen L.; Gao, Q.; Yang, L. Determination of Promethazine and Chlorpromazine in Poultry Eggs by High Performance Liquid Chromatography Tandem Mass Spectrometry. *The Food Ind.*, **2020**. *41*, 299–302.

49. Sun, L.; Zhang, L.; Xu, Q.; Wang, S.; Wang, X. Determination of ten sedative residues in pork and kidney by ultra performance liquid chromatography- tandem mass spectrometry. Chin. J. of Chromatogr., **2010**, *28*, 38–42.

50. Li, Z.;Li, H.;Ma, Y.;Wang, S.;Ren, N.;Guo, W.;Guo, C.;Li, Y. High Performance Liquid Chromatography-Tandem Mass Spectrometry Detection of Promethazine in Animal-Derived Foods. *Food Sci.*, **2019**, *40,* 320–324.

51. Wang, J.; Ye, J.; Zhong, S.; Wang, X.; Chen Q. Determination of Antihistamine R esidues in Livestock and Poultry Products by Ultra Performance Liquid Chromatography－Quadrupole－Time of Flight Mass Spectrometry. *Sci. Technol. of Food Ind.*, **2021**, *42,* 250–256, 264.

52. Liang, Y.; Li, Y.; Guo, X.; Li, R.; Gao, L.; Wang, J.; Fu, G.; Wan, Y. Determination of chlorpromazine, promethazine, and vancomycin in pork product by liquid chromatography-mass spectrometry. *Cereal Feed Ind.*, **2016**, *346*, 69–73.

**Table S2.** Original Data of Recovery and precision of spiked blank samples.

| Tissues | Analyte | Concentration（μg/kg） | | | | Recovery ($\overline{X}$±S.D)(n=6) (%) | | | Intra-day RSD（%）(n=6) | | | Inter-day RSD |
|---|---|---|---|---|---|---|---|---|---|---|---|---|
| | | Spiked | Found（$\overline{X}$, n=6） | | | Batch I | Batch II | Batch III | Batch I | Batch II | Batch III | （%） |
| **Muscle** | PMZ | 0.1 | 0.077 | 0.082 | 0.082 | 77.13±5.44 | 81.73±9.12 | 82.35±8.82 | 7.05 | 11.16 | 10.71 | 9.86 |
| | | 5 | 5.00 | 4.58 | 4.72 | 100.00±2.82 | 91.62±2.92 | 94.47±4.51 | 2.82 | 3.18 | 4.78 | 5.13 |
| | | 50 | 52.33 | 47.15 | 50.08 | 104.55±5.98 | 94.37±3.56 | 100.27±3.84 | 5.72 | 3.78 | 3.83 | 6.12 |
| | PMZSO | 0.1 | 0.10 | 0.10 | 0.099 | 102.95±7.21 | 103.47±8.33 | 99.22±10.31 | 7.00 | 8.06 | 10.39 | 8.26 |
| | | 5 | 4.64 | 5.31 | 5.17 | 92.82±1.97 | 106.33±3.20 | 103.28±7.97 | 2.12 | 3.01 | 7.72 | 7.62 |
| | | 50 | 43.12 | 45.23 | 43.95 | 86.25±2.08 | 90.45±2.99 | 87.88±2.84 | 2.42 | 3.30 | 3.24 | 3.49 |
| | Nor$_1$PMZ | 0.5 | 0.52 | 0.45 | 0.47 | 103.70±7.89 | 90.03±7.53 | 94.33±8.84 | 7.61 | 8.36 | 9.37 | 10.02 |
| | | 5 | 4.75 | 4.81 | 5.04 | 95.00±6.20 | 96.10±3.50 | 100.73±4.97 | 6.52 | 3.64 | 4.93 | 5.53 |
| | | 50 | 45.92 | 55.58 | 53.70 | 91.85±2.97 | 111.17±3.66 | 107.43±6.38 | 3.24 | 3.29 | 5.95 | 9.27 |
| **Liver** | PMZ | 0.1 | 0.10 | 0.10 | 0.097 | 101.02±10.90 | 104.15±7.39 | 96.72±6.09 | 10.79 | 7.10 | 6.29 | 8.42 |
| | | 5 | 5.37 | 5.13 | 4.35 | 107.17±2.48 | 102.72±8.20 | 87.08±4.55 | 2.32 | 7.99 | 5.22 | 10.42 |
| | | 50 | 44.37 | 45.42 | 44.53 | 88.75±4.44 | 90.82±5.07 | 89.10±3.87 | 5.00 | 5.58 | 4.35 | 4.84 |
| | PMZSO | 0.1 | 0.098 | 0.10 | 0.098 | 97.52±7.88 | 101.22±5.12 | 97.88±9.62 | 8.08 | 5.06 | 9.83 | 7.58 |
| | | 5 | 4.58 | 5.01 | 4.54 | 91.52±6.05 | 100.23±3.94 | 90.72±4.96 | 6.61 | 3.93 | 5.46 | 6.89 |
| | | 50 | 50.02 | 48.23 | 52.20 | 100.02±4.42 | 96.48±5.13 | 104.33±1.86 | 4.42 | 5.31 | 1.78 | 5.10 |
| | Nor$_1$PMZ | 0.5 | 0.49 | 0.48 | 0.47 | 97.48±8.35 | 95.41±6.61 | 94.17±6.01 | 8.56 | 6.92 | 6.39 | 7.47 |
| | | 5 | 4.57 | 4.76 | 4.93 | 91.28±5.03 | 95.28±8.94 | 98.50±6.85 | 5.51 | 9.38 | 6.95 | 7.76 |
| | | 50 | 47.32 | 49.33 | 50.40 | 94.63±3.85 | 98.67±4.29 | 100.88±3.60 | 4.07 | 4.35 | 3.57 | 4.59 |
| **Kidney** | PMZ | 0.1 | 0.086 | 0.088 | 0.094 | 86.18±4.29 | 88.05±2.53 | 94.36±7.33 | 4.98 | 2.87 | 7.76 | 6.70 |
| | | 5 | 4.27 | 4.48 | 4.22 | 85.48±5.05 | 89.63±4.34 | 84.30±3.63 | 5.91 | 4.84 | 4.30 | 5.45 |
| | | 50 | 45.88 | 44.78 | 45.43 | 91.80±2.77 | 89.60±5.68 | 90.83±1.68 | 3.02 | 6.34 | 1.84 | 4.02 |
| | PMZSO | 0.1 | 0.10 | 0.095 | 0.097 | 100.22±10.67 | 94.52±9.60 | 97.07±6.56 | 10.16 | 10.65 | 6.76 | 9.14 |
| | | 5 | 5.02 | 5.23 | 5.10 | 100.60±4.31 | 104.53±5.88 | 101.95±3.11 | 4.29 | 5.63 | 3.05 | 4.55 |
| | | 50 | 47.80 | 49.58 | 45.47 | 95.62±1.70 | 99.17±4.00 | 90.90±2.19 | 1.77 | 4.03 | 2.04 | 4.58 |
| | Nor$_1$PMZ | 0.5 | 0.47 | 0.48 | 0.50 | 93.75±6.36 | 95.92±11.94 | 99.15±5.35 | 6.79 | 12.45 | 5.39 | 8.62 |
| | | 5 | 5.12 | 4.93 | 5.22 | 102.42±7.24 | 97.40±10.32 | 104.30±6.13 | 7.07 | 10.59 | 5.88 | 7.86 |
| | | 50 | 52.18 | 47.95 | 49.70 | 104.22±5.79 | 95.92±6.93 | 99.32±2.69 | 5.56 | 7.23 | 2.71 | 6.32 |

| | | | | | | | | | | | | |
|---|---|---|---|---|---|---|---|---|---|---|---|---|
| **Fat** | PMZ | 0.1 | 0.097 | 0.10 | 0.096 | 96.87±7.32 | 100.37±7.60 | 96.13±9.16 | 7.55 | 7.57 | 9.53 | 7.63 |
| | | 5 | 4.88 | 5.03 | 5.20 | 97.62±3.58 | 100.50±3.12 | 104.05±3.30 | 3.67 | 3.10 | 3.17 | 4.12 |
| | | 50 | 52.35 | 48.83 | 50.33 | 104.55±6.95 | 97.63±5.47 | 100.53±5.67 | 6.65 | 5.60 | 5.64 | 6.40 |
| | PMZSO | 0.1 | 0.094 | 0.10 | 0.095 | 94.02±6.79 | 100.55±10.52 | 94.73±8.52 | 7.22 | 10.46 | 9.00 | 9.07 |
| | | 5 | 4.23 | 4.43 | 4.90 | 84.65±3.88 | 88.66±3.74 | 97.85±5.20 | 4.59 | 4.22 | 5.32 | 7.73 |
| | | 50 | 46.30 | 45.48 | 46.40 | 92.56±3.32 | 90.93±4.65 | 92.82±3.00 | 3.59 | 5.11 | 3.23 | 4.79 |
| | $Nor_1PMZ$ | 0.1 | 0.10 | 0.096 | 0.10 | 100.47±9.91 | 96.07±8.68 | 101.38±11.00 | 9.86 | 9.04 | 10.85 | 9.68 |
| | | 5 | 5.00 | 5.22 | 5.26 | 100.13±6.51 | 104.17±7.68 | 105.38±5.49 | 6.50 | 7.37 | 5.21 | 6.37 |
| | | 50 | 49.38 | 47.88 | 43.43 | 98.73±4.93 | 92.25±7.07 | 86.88±6.87 | 4.99 | 7.66 | 7.90 | 7.53 |
